# Temporal avoidance as a means of reducing competition between sympatric species

**DOI:** 10.1098/rsos.230521

**Published:** 2023-05-24

**Authors:** Marta Maziarz, Richard K. Broughton, Kristina B. Beck, Robert A. Robinson, Ben C. Sheldon

**Affiliations:** ^1^ Edward Grey Institute, Department of Biology, Oxford University, Oxford OX1 3SZ, UK; ^2^ Polish Academy of Sciences, Museum and Institute of Zoology, Wilcza 64, Warsaw 00-679, Poland; ^3^ UK Centre for Ecology & Hydrology, Wallingford OX10 8BB, UK; ^4^ British Trust for Ornithology, The Nunnery, Thetford IP24 2PU, UK

**Keywords:** interspecific competition, interference, foraging behaviour, mixed-species groups, social hierarchy, sympatric woodland species

## Abstract

Human activity has modified the availability of natural resources and the abundance of species that rely on them, potentially changing interspecific competition dynamics. Here, we use large-scale automated data collection to quantify spatio-temporal competition among species with contrasting population trends. We focus on the spatial and temporal foraging behaviour of subordinate marsh tits *Poecile palustris* among groups of socially and numerically dominant blue tits *Cyanistes caeruleus* and great tits *Parus major*. The three species exploit similar food resources in mixed groups during autumn–winter. Using 421 077 winter recordings of individually marked birds at 65 automated feeding stations in Wytham Woods (Oxfordshire, UK), we found that marsh tits were less likely to join larger groups of heterospecifics, and they accessed food less frequently in larger groups than in smaller ones. Marsh tit numbers within groups declined throughout the diurnal and winter periods, while the number of blue and great tits increased. However, sites that attracted larger groups of these heterospecifics also attracted more marsh tits. The results suggest that subordinate species exhibit temporal avoidance of socially and numerically dominant heterospecifics, but have limited ability for spatial avoidance, indicating that behavioural plasticity enables only a partial reduction of interspecific competition.

## 1. Introduction

Interspecific competition for resources (e.g. food and shelter) is limited when the resources are abundant and consumers occur at low densities [1], such as in primeval conditions least modified by humans [2,3]. Human activity often modifies the quality and quantity of key resources, influencing the abundance of the species that rely on them [4–6] and potentially altering the interspecific interactions in rapidly changing anthropogenic habitats [5].

Interspecific competition for limited resources may drive population declines of subordinate species in the presence of dominant ones [1,5]. However, confirming a causal link between competition and population dynamics can be challenging, particularly when species compete indirectly through resource depletion [1,7]. Nonetheless, interference competition, such as aggression or exclusion of subordinate individuals by dominants, may be a straightforward sign of negative interactions, and this can be readily observed in mixed-species groups [1,8,9].

Aggregating in mixed-species groups can be beneficial for individuals in locating food sources or reducing the risk of predation, but only when the benefits offset the costs [8]. The costs could involve increased interference competition, which may be unequally distributed among conspecifics and heterospecifics, depending on their social status within the group [10,11]. The lowest-ranked individuals often suffer the greatest costs of competition, so may be less willing to join groups of conspecifics of a higher social status, and least likely to associate with socially and numerically dominant heterospecifics that effectively control access to food [12,13].

When inference competition increases, individuals usually forage less efficiently, so their feeding rates can indicate the intensity of negative interactions between group members [9]. As interspecific competition is likely to be greatest in large groups of dominants, to maximize their own fitness, the low-ranking individuals of subordinate species should join groups of an optimum size and composition, and avoid large aggregations of dominant heterospecifics in particular [8,12]. Such individuals could reduce interspecific competition by, for example, adjusting their food-handling times, foraging strategies or avoiding congested feeding sites [12,14–18].

Tits (Paridae) are small songbirds of temperate or boreal woodlands and a model group for studying interspecific interactions, notably in their mixed-species flocks [9]. In Europe, these flocks include marsh tits *Poecile palustris* and more abundant blue tits *Cyanistes caeruleus* and great tits *Parus major* [6,19–21]. In autumn–winter, these species often forage together and exploit similar food (seeds and invertebrates), forming fission–fusion societies which vary in size and composition over space and time [13,22,23]. Like other vertebrate taxa [10,24], mixed-species groups of tits exhibit a social dominance hierarchy. Marsh tits are typically subordinate to blue tits and great tits, and immature marsh tits represent the lowest-ranked individuals relative to adult conspecifics or any heterospecifics [20,25].

In the modified landscape of Britain, where highly fragmented and ecologically degraded woodlands are sparsely distributed within a primarily farmland matrix [26,27], interspecific competition for food with blue tits and great tits has been proposed as a potential factor in the long-term population decline of marsh tits (*ca* 80% since the mid-1960s) [28,29]. Aggressive supplanting of marsh tits by these dominant heterospecifics has previously been observed at feeders and among birds foraging in trees [20,30,31]. However, there has been no empirical study of the marsh tit's social behaviour towards varying numbers of heterospecifics or conspecifics, which could signal differing levels of competition. The three tit species are, therefore, an ideal model group to explore interference competition in human-transformed landscapes, with important conservation implications for declining species [4,32,33].

Here we examine the spatial and temporal components of interference competition between subordinate and dominant bird species in mixed groups. We determine the likelihood of marsh tits joining a group and their feeding rates in relation to varying numbers of heterospecifics and conspecifics, reflecting a range of costs and benefits of flocking [8]. We test the responses of individual marsh tits according to their social status as reflected by age class of subordinate immatures and dominant adults [25]. Finally, we explore changes in group sizes to test the temporal and/or spatial avoidance by marsh tits of large groups of dominant heterospecifics. We discuss the consequences of increased interference competition for declining species in human-transformed environments.

## 2. Methods

### 2.1. Study species

The marsh tit is a small (*ca* 10 g), sedentary songbird that is a specialist of mature broadleaved woodlands in Europe and East Asia [34]. In Britain, casual groups of around three marsh tits, but sometimes up to 10, occupy large, partially overlapping home ranges during winter, averaging 31 ha each, which are loosely centred on spring territories [35]. Seeds are an important part of the marsh tit diet, and are habitually cached for later consumption [36].

Blue tits (*ca* 10 g) and great tits (*ca* 20 g) occur in similar woodland habitats alongside marsh tits, but breed at higher densities and are more generalist, and also occur in a wider range of habitats outside woodlands [34,36]. Blue tits and great tits can be mobile in winter, and move to suburban areas to exploit supplementary food [7,37]. They have broadly similar winter diets to marsh tits, but do not cache seeds [36].

Previous studies have demonstrated a clear dominance hierarchy between the three tit species across their ranges, where blue and great tits are socially dominant to marsh tits [19,20,38,39]. This dominance can take the form of control of resources as a result of passive avoidance by the subordinate species, and also aggressive displacement and supplanting attacks by the dominants within mixed flocks.

### 2.2. Data collection

The study was conducted in the 385 ha Wytham Woods, near Oxford, UK (51°46′ N, 01°20′ W) [40]. Data collection involved marking of nestlings in spring or trapping and marking of full-grown individuals during autumn and winter using mist-nets at feeders [22,41]. Over 90% of tits in the study area were marked with individually numbered leg-rings and plastic rings containing a uniquely identifiable passive integrated transponder (PIT) tag [41]. Marsh tits were aged based on their ringing record as nestlings or on plumage features during autumn and winter trapping, being classed as immatures (less than 1 year old) or adults [42].

Individuals were recorded at 65 automated feeding stations baited with sunflower seeds, distributed *ca* 250 m apart in a grid across Wytham Woods [43]. The feeding stations were equipped with radio-frequency identification (RFID) antennas and data loggers that provided a timestamp of individual visits to feeders (Dorset ID, The Netherlands). Data were collected over three winters: from 3 December 2011 to 25 February 2012, 1 December 2012 to 23 February 2013 and 30 November 2013 to 22 February 2014. Each feeding station had two access points (each with an RFID antenna) to minimize queuing as individuals collected seeds on rapid visits. All feeding stations opened simultaneously only on weekends (Saturday and Sunday), providing food continuously during daylight hours, with PIT-tags on arriving birds being logged every one third of a second. More than 99% of visits by tagged individuals were detected, providing synchronous snapshots of association patterns in the mixed-species groups on 13 sampling weekends each winter [43]. The disturbance of birds at feeders by the public was negligible as access to Wytham Woods requires walking permits, which limits the number of people entering the woods. Also, visitors are required to keep to major paths in restricted areas away from feeders, and are not permitted before 10 am. Visitor numbers are generally low during the winter, when data collection occurred.

Group membership was defined using a machine learning algorithm based on Gaussian mixture models, which automatically assigned bursts of high bird activity, separated by periods of low activity, into individual ‘flocking events’ [44]. The method returns a matrix of flocking events and the individual birds assigned to each of them, with an associated time and date stamp. Each flocking event was specific to a single feeder, on a specific day, and lasting for a specific duration.

To improve computation performance, we used information collected across all feeders only on the first day of each weekend (Saturday). Additionally, to reduce possible false negatives of bird absence due to equipment malfunction, we included only those feeding stations at which bird activity was recorded for a minimum of 6 hours. We used data only from the main period of bird activity from 07.00 to 17.00 (GMT; 10 h in total), as earlier or later recordings were scarce (*ca* 0.3%).

### 2.3. Data analysis

We obtained information from 149 346 flocking events involving 60–65 active feeding stations in each winter, giving 421 077 records of an individual marsh tit's presence or absence. For each flocking event the dataset comprised: the feeding station identity, date and time (hour and minute) of when the event began, presence or absence of an individual marsh tit at the feeder visited on that given day, age (immature or adult), the number of conspecifics and the combined number of heterospecifics (blue tits and/or great tits).

Feeding rates of individual marsh tits were calculated from the number of visits to feeding stations during each of the 105 641 flocking events when marsh tits were present. We grouped the continuous recordings of individuals within 20 s sections, which were assigned to single visits lasting for up to 20 s during a flocking event [45]. To account for different durations of flocking events, which ranged between *ca.* 1 and 112 min, feeding rates were initially calculated as the actual number of marsh tit visits per minute of a flocking event. When a flocking event lasted for less than a full minute (5.6% of 105 641 events), we used the actual number of visits. The beginning and end of each flocking event was given with a resolution of 1 min, so feeding rates could not be assessed with a finer time resolution. All feeding rates were then multiplied by 10 to obtain the rates per 10 min, which helped to improve the clarity for interpretation.

We were interested in the combined effect of dominant blue and great tits on the behaviour of subordinate marsh tits. Therefore, we used the summed numbers of blue and great tits present in a flocking event, indicating the group sizes of heterospecifics. The group size of marsh tits was defined as the number of conspecifics recorded together during a flocking event, within a single- or mixed-species group (respectively 10% and 90% of 105 641 flocking events).

To assess temporal and spatial changes in group sizes of marsh tits and their heterospecifics, we calculated the mean hourly values derived from the flocking events. Mean hourly values of group size were assessed separately for each feeding station on a given Saturday and year. To test whether marsh tit group sizes were smaller at feeding sites occupied by larger groups of the dominant species, for each feeding station we calculated the mean annual (winter period) group sizes of marsh tits and the heterospecifics. Mean annual values for each feeding station were obtained from the 13 mean daily group sizes each winter, which were derived from the hourly means. Using the mean hourly or annual group sizes simplified the model structure and improved the performance of processing the large dataset.

### 2.4. Statistical analyses

#### 2.4.1. Likelihood of joining a group and feeding rates of marsh tits

We performed all statistical analyses in R v. 4.1.0 [46]. To test the likelihood of an individual marsh tit joining a group, we used a generalized linear mixed model (GLMM) with a binomial error structure (package ‘lme4’ [47]). The model contained fixed predictors of the number of group members in a flocking event, separately for marsh tits and heterospecifics (blue and great tits combined), and the marsh tit's age class (immature or adult). To investigate the effects of temporally changing group sizes of heterospecifics [23] from the possible unrelated temporal shifts in marsh tit activity, in the model we also added three (fixed) temporal covariates: hour (from 07.00 to 17.00 GMT), week (1–13) and year (2011–2012, 2012–2013 or 2013–2014).

We presumed that the likelihood of marsh tit presence would change in a nonlinear fashion with the number of group members (heterospecifics or conspecifics) and also with hour or week, so we tested quadratic effects of these four numerical predictors. We expected an optimum likelihood of marsh tit presence at moderate group sizes of heterospecifics or conspecifics, indicative of the trade-off between the benefits and costs of flocking [8]. For the temporal covariates, we expected a greater likelihood of marsh tit presence at midday and in midwinter as birds discovered the feeding sites and later moved away to roost each day and to occupy territories at the end of winter.

Additionally, the model contained three interaction terms between year and the number of heterospecifics, year and the number of conspecifics, and the number of heterospecifics and conspecifics in a group. The interaction terms considered the variation in marsh tit responses to the annually changing numbers of group members and varying proportions of heterospecifics and conspecifics in a group [22,48]. The model incorporated random intercepts of marsh tit and feeding station identities to account for the non-independent responses of the same individuals in subsequent flocking events, and their spatial variation. We standardized (scaled) all numeric predictors (mean-centred and divided by standard deviations).

To test for changes in marsh tit feeding rates in relation to the number of heterospecifics or conspecifics in a group, we used a linear mixed model (LMM; package ‘lme4’) fitted with restricted maximum likelihood (REML). The model contained the same set of fixed predictors and random intercepts as described above. We used standardized (mean-centred and divided by the standard deviation) feeding rates as a response variable.

#### 2.4.2. Temporal and spatial changes in group sizes

We tested the nonlinear changes of mean hourly group sizes with general additive models (GAM) and a Gaussian distribution function (packages ‘nlme’ and ‘mgcv’ [49,50]). We produced two separate models with a response of the mean hourly group size of marsh tits or the mean hourly group size of heterospecifics (both scaled), and the same set of temporal (hour, week, year) and spatial (feeding station identity) predictors.

We assessed the change in group sizes during a day and within a winter period for each year independently. Therefore, both models included a fixed effect of year and interaction terms between hour and year, or week and year, with a thin plate regression spline (a default smoothing term). To test whether the temporally changing group sizes of marsh tits indicated their avoidance of large groups of heterospecifics, we added a smoothed term (thin plate regression spline) of the mean hourly number of heterospecifics in a flock as a predictor of marsh tit group size. To account for spatial variation in mean hourly group sizes, we used a smoothing term with a random effect for feeding station identity. We applied an automatic selection of the optimal number of knots by cross-validation and fitted both models with REML.

To test whether groups of marsh tits were smaller at feeding stations that were visited by larger groups of heterospecifics, suggesting spatial avoidance, for each feeding station we assessed the relationship between the mean annual group sizes of marsh tits and their heterospecifics. We found significant spatial autocorrelation in mean annual group sizes of birds between feeding stations in two winter periods, 2011–2012 and 2012–2013 (Mantel test, marsh tits versus heterospecifics respectively: *r* = 0.09–0.17 versus 0.07–0.11, *p* ≤ 0.05; package ‘ade4’ [51–54]). To account for this non-random spatial distribution of group sizes, we used a Gaussian spatial correlation model structure with Euclidean distances and a nugget effect [49]. We standardized (mean-centred and divided by standard deviations) mean annual group sizes of marsh tits and heterospecifics and ran a generalized least-squares (GLS) model fitted with REML (package ‘nlme’) and an applied correlation structure within a year level.

We used the combined numbers of blue and great tits in all analyses, as the responses of marsh tits were alike to both heterospecifics, which numerically dominated marsh tits at Wytham Woods (electronic supplementary material, figures S1–S5, electronic supplementary material, tables S1–S3).

## 3. Results

### 3.1. Likelihood of joining a group and feeding rates of marsh tits

**Figure 1.**
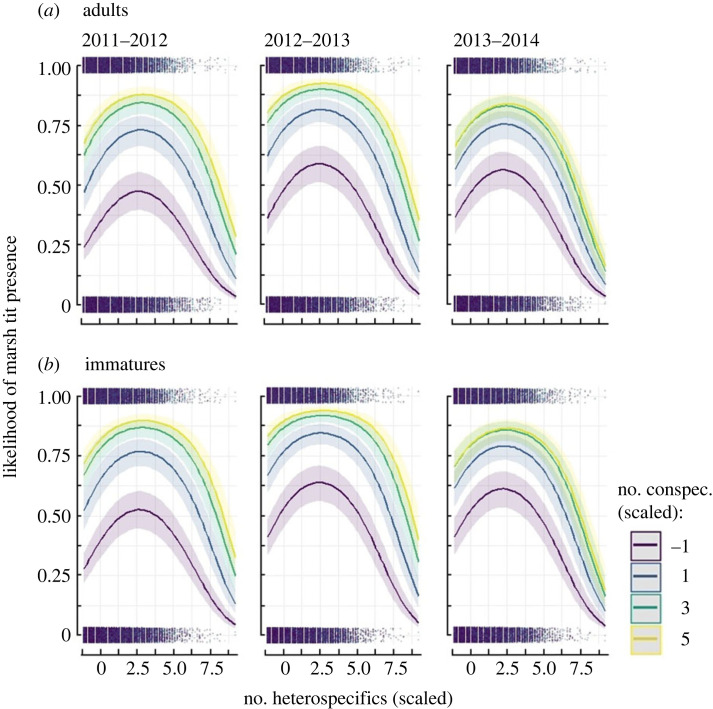
The likelihood of (*a*) adult and (*b*) immature marsh tits joining a flock at feeding stations visited on given days in relation to the standardized number of heterospecifics (blue and great tits combined) and conspecifics in 149 346 flocking events. Mean (s.d.) numbers of heterospecifics and conspecifics were respectively: 7.2 (6.24) and 1.4 (1.44) in 2011–2012, 5.6 (5.62) and 0.9 (0.92) in 2012–2013, and 5.3 (4.95) and 0.8 (0.82) in 2013–2014. Lines are mean trends, shades are 95% confidence intervals of fixed predictions on the population level obtained from binomial GLMM (table 1), jittered dots are presence (1) or absence (0) of individual marsh tits (*n* = 209) in flocking events.

The likelihood of marsh tits joining a group initially increased with the number of heterospecifics, but it declined sharply when groups of heterospecifics exceeded 25 blue tits and/or great tits combined (figure 1, table 1). Nevertheless, the likelihood of marsh tit presence was consistently higher if flocks also contained conspecifics. A larger number of conspecifics in a flock resulted in an increase in the likelihood of marsh tit presence, largely irrespective of the number of heterospecifics in the same flock (figure 1). Consequently, the effect of an interaction term between the number of heterospecifics and conspecifics was small if nearly statistically significant due to very large sample sizes (table 1).

**Table 1.** Results of binomial generalized linear mixed model (GLMM) and linear mixed model (LMM; fitted by REML), testing the likelihood of individual marsh tits (*n* = 209) joining a flock and their feeding rate in relation to the individual's age (adult or immature), the number (*n*) of heterospecifics (blue and great tits combined) and/or conspecifics in a flock, hour (from 07.00 to 17.00 GMT), week (1–13) and year of a flocking event. Shown are standardized coefficients (*β*) and 95% CI (confidence intervals). NA, not applicable. GLMM versus LMM respectively: $R_{\mathrm{cond}.}^{2}=0.52$ versus 0.23, $R_{\mathrm{marg}.}^{2}=0.09$ versus 0.13.

variable	likelihood of presence			feeding rate		
fixed effects	*β*	CI 2.5%	CI 97.5%	*β*	CI 2.5%	CI 97.5%
intercept	−0.05	−0.38	0.27	−0.07	−0.14	−0.01
year (2012–2013)	0.56	0.54	0.59	−0.02	−0.04	−0.01
year (2013–2014)	0.41	0.38	0.44	−0.15	−0.18	−0.13
age (immatures)	0.20	0.17	0.24	0.11	0.10	0.13
*n* heterospecifics (linear)	0.41	0.39	0.42	−0.29	−0.30	−0.28
*n* heterospecifics (quadratic)	−0.07	−0.08	−0.07	0.03	0.03	0.04
*n* conspecifics (linear)	0.53	0.51	0.54	−0.14	−0.15	−0.13
*n* conspecifics (quadratic)	−0.05	−0.06	−0.05	0.02	0.02	0.03
hour (linear)	−0.17	−0.18	−0.17	−0.15	−0.16	−0.15
hour (quadratic)	−0.07	−0.08	−0.07	−0.01	−0.02	−0.01
week (linear)	−0.38	−0.39	−0.37	−0.11	−0.11	−0.10
week (quadratic)	−0.08	−0.09	−0.07	−0.04	−0.05	−0.04
*n* heterospec. x year (2012–2013)	−0.03	−0.05	−0.02	0.00	−0.01	0.01
*n* heterospec. x year (2013–2014)	−0.07	−0.09	−0.05	−0.03	−0.04	−0.02
*n* conspec. x year (2012–2013)	0.01	−0.01	0.03	−0.02	−0.03	0.00
*n* conspec. x year (2013–2014)	−0.11	−0.13	−0.08	−0.04	−0.05	−0.02
*n* heterospec. x *n* conspec.	0.01	0.00	0.02	0.05	0.04	0.05
random effects	variance	s.d.		variance	s.d.	
bird ID (intercept)	1.55	1.24		0.08	0.28	
feeder ID (intercept)	1.36	1.17		0.03	0.18	
residual	NA	NA		0.80	0.90	

The patterns of marsh tit presence in relation to the number of group members were consistent between years and age classes (figure 1), although the likelihood generally increased over consecutive winters, and immature marsh tits were more likely to be present than adults (table 1). Additionally, the likelihood of marsh tit presence in a group increased in the morning and early in the season, but then declined in the afternoon and later in the winter; the negative effects of the two temporal variables were distinct, as the linear coefficients were relatively large at narrow 95% confidence intervals (CIs; table 1).

**Figure 2.**
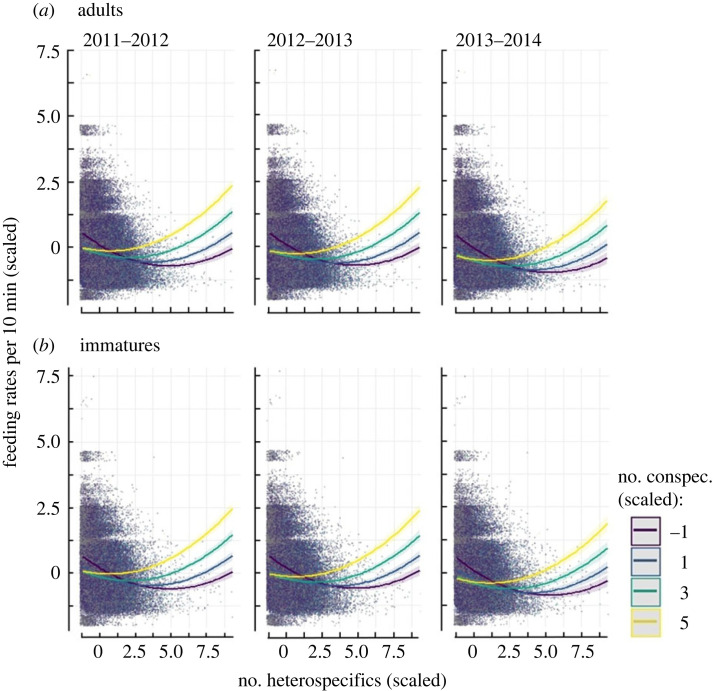
The feeding rates of (*a*) adult and (*b*) immature marsh tits in relation to the standardized number of heterospecifics (blue and great tits combined) and conspecifics during 105 641 flocking events. Lines are mean trends, shading is 95% confidence intervals of fixed predictions on the population level obtained from LMM (table 1), dots are feeding rates of individual marsh tits.

Marsh tit feeding rates during flocking events initially declined with the number of heterospecifics if conspecifics were absent in the same groups (figure 2). When conspecifics were present, the feeding rates of marsh tits were initially low but the declines were less acute with increasing numbers of heterospecifics, in contrast to the flocks without conspecifics (figure 2). However, the coefficient of the interaction term between the number of heterospecifics and conspecifics was low, although the 95% CIs were narrow and non-overlapping with 0 due to large sample sizes (table 1). The feeding rates increased again in the largest groups, i.e. those exceeding 40 heterospecifics, but these were rare (less than 0.6% of 149 346 flocking events) and so the feeding rates may be inflated.

The patterns of feeding rates in relation to the number of heterospecifics and conspecifics were alike between years and age classes (figure 2), despite feeding rates falling since 2011–2012, and they were generally lower for adult than immature marsh tits (table 1). Overall, then, the feeding rates of marsh tits generally declined after an initial increase during the day and within a season (table 1).

### 3.2. Temporal and spatial changes in group sizes

Groups of marsh tits contained a mean 1.8 individuals (s.d. = 0.96, range = 1–9, *n* = 105 641 flocking events), less than one third of the average size of combined groups of blue and great tits (mean 6.2, s.d. = 5.32, range = 1–60, *n* = 135 943 flocking events).

**Figure 3.**
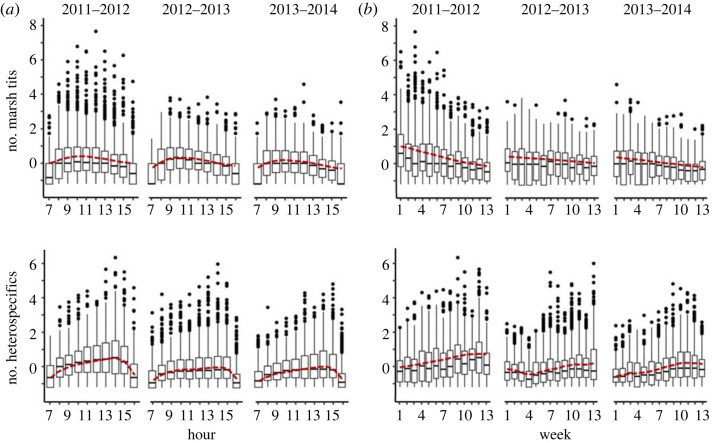
(*a*) Hourly and (*b*) weekly changes in standardized (scaled) group sizes of marsh tits and heterospecifics (blue and great tits combined) in three consecutive winters. Boxes are 25–75% intervals, whiskers are ranges within 1.5 × 25–75%, black dots are outliers, horizontal lines are medians, red dashed lines are mean trends obtained from GAMs (table 2).

**Figure 4.**
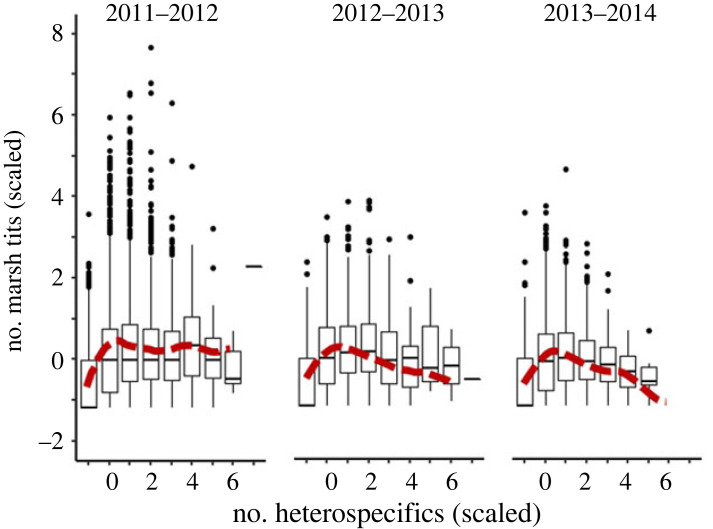
Standardized mean hourly group size of marsh tits in relation to the respective group size of heterospecifics (blue and great tits combined) in flocks in three consecutive winters. Boxes are 25–75% intervals, whiskers are ranges within 1.5 × 25–75%, black dots are outliers, horizontal lines are medians, red dashed lines are mean trends obtained from GAMs (table 2).

The mean hourly group sizes fluctuated during the day and within the winter, with a temporal divergence between marsh tits and heterospecifics each year (figure 3, table 2). The mean hourly group size of marsh tits typically peaked at 09.00–11.00 in the morning and declined later in the day, whereas the group size of blue and great tits peaked at 14.00–15.00 (figure 3*a*). Similarly, marsh tit group sizes declined over the winter, coinciding with increased group sizes of heterospecifics (figure 3*b*). Furthermore, the mean hourly group sizes of marsh tits initially increased but then usually declined when heterospecifics became abundant (figure 4; table 2).

**Table 2.** Results of general additive models (GAMs) testing hourly and weekly changes of mean hourly group sizes of marsh tits versus combined blue and great tits (fitted by REML). Smoothed term with a random effect accounting for spatial variation. Effective degrees of freedom (edf) is a measure of nonlinearity of the smoother. NA, not applicable. $R_{\mathrm{adj}.}^{2}$ of models which tested the changes in group size of marsh tits and heterospecifics were 0.42 and 0.57 respectively, *n* = 17 520 hourly means.

variables	marsh tits				blue and great tits combined			
fixed term:	*β*	s.e.	*t*	*p*	*β*	s.e.	*t*	*p*
intercept	0.00	0.06	0.0	0.976	0.13	0.08	1.6	0.101
year 2012–2013	−0.10	0.01	−6.6	4×10^−11^	−0.38	0.01	−31.4	<2×10^−16^
year 2013–2014	−0.26	0.02	−17.0	<2×10^−16^	−0.44	0.01	−34.8	<2×10^−16^
smooth terms:		edf	*F*	*p*		edf	*F*	*p*
*thin plate regression spline*								
hour by year:								
2011–2012		5.6	32.9	<2×10^−16^		8.4	227.3	<2×10^−16^
2012–2013		6.3	46.6	<2×10^−16^		8.4	120.1	<2×10^−16^
2013–2014		6.1	35.1	<2×10^−16^		8.3	111.9	<2×10^−16^
week by year:								
2011–2012		6.6	151.2	<2×10^−16^		8.7	110.1	<2×10^−16^
2012–2013		1.0	124.9	<2×10^−16^		8.7	71.8	<2×10^−16^
2013–2014		4.8	47.1	<2×10^−16^		6.1	121.2	<2×10^−16^
*n* heterospecifics by year:								
2011–2012		8.0	93.1	<2×10^−16^		NA	NA	NA
2012–2013		7.7	56.9	<2×10^−16^		NA	NA	NA
2013–2014		7.4	54.2	<2×10^−16^		NA	NA	NA
*random effects*								
feeder ID		63.3	112.4	<2×10^−16^		63.7	246.5	<2×10^−16^

Despite a large spatial variation in mean hourly group sizes (table 2), sites visited by larger groups of heterospecifics were also typically visited by larger groups of marsh tits (GLS, *β* = 0.33, 95% CI = 0.20–0.46).

## 4. Discussion

The results support the hypothesis of interference competition between subordinate marsh tits and the dominant heterospecifics, i.e. blue tits and great tits. The likelihood of marsh tits joining a group declined sharply when heterospecifics became abundant, exceeding 25 individuals. Additionally, marsh tit feeding rates tended to decline with increasing numbers of heterospecifics, suggesting reduced foraging efficiency [9], albeit with the uncommon exception of the largest groups. Group sizes of marsh tits and heterospecifics diverged temporally, both during the day and within each winter season. The dominant heterospecifics were most abundant at feeding stations during the afternoons and in late winter, whereas largest groups of marsh tits were most common in the mornings and in early winter. As the feeding stations provided a continuous supply of food during daylight hours on weekends (see also [13]), food was ad libitum, and so the observed patterns of activity should not involve exploitation competition, but rather interference. Supplanting of marsh tits by dominant and numerous heterospecifics (blue and great tits) is common at feeders [19,20,38], and probably occurred also in our study. Thus, interference competition seemed the most likely explanation for the marsh tits' reduced foraging activity and apparent avoidance of large groups of heterospecifics.

Despite the artificial system of fixed feeding stations that supplied food on weekends as a recurring pulsed resource, this arrangement imitated natural conditions of clumped and ephemeral food resources, such as masting trees in winter [55,56]. The long gaps between weekends, when food was unavailable, would prevent birds becoming reliant on this supplementary food, and limit influences on their social behaviour. The findings are also consistent with other studies involving the same or other taxa, and including natural food sources [9]. The flocking behaviour of marsh tits follows the expected patterns of group formation and optimal group size in animals [8–11]. The increasing likelihood of birds joining a group suggests initial benefits outweighing the costs, but the declining likelihood at larger group sizes of heterospecifics indicates that the costs of flocking become dominant.

The temporal divergence in group sizes of the different tit species seemed to be linked to the interspecific avoidance by subordinate marsh tits of the dominant blue and great tits, as the likelihood of marsh tit presence and their group sizes declined when heterospecifics became abundant. These negative relationships remained significant even after accounting for the temporal changes in the foraging activity of marsh tits at feeders. Moreover, the declining group sizes of marsh tits during each winter seemed little affected by the birds’ mortality or abundance. The apparent mortality of marsh tits averaged less than 5% over each winter (electronic supplementary material, table S4), yet their mean daily group sizes still declined within a season (while increasing for blue tits and great tits; electronic supplementary material, table S5), even when weekly fluctuations in abundance were considered (electronic supplementary material, figure S1). A similar temporal divergence in foraging activity between subordinate and dominant species has been observed in other taxa, including insects [57,58], fish [12], mammals and other birds [14,15,17,59], indicating a general response for reducing interference competition.

By concentrating their foraging early in the day, marsh tits might cache some food and switch to these stored resources later in the day, when heterospecifics dominate access to the feeding sites. Caching food is a frequent strategy of some animals, which may buffer individuals from periods of unpredictable weather and limited access to food, but could also help subordinates avoid interspecific competition [60,61]. However, caching is a short-term solution for marsh tits, as cached food is usually retrieved within a few hours or days [62]. Thus, when cached food depletes, subordinates might face more acute inference competition, possibly being displaced by dominant species to suboptimal foraging sites, as found in other mixed-species groups [9,10,63].

Behavioural plasticity may allow subordinate species to employ temporal or spatial avoidance of dominant competitors, facilitating cohabiting species to use the same resources. As any behavioural plasticity has its limitations, however, sufficient avoidance of interference competition could become more difficult, especially for habitat specialists, such as marsh tits, which have a narrower niche and are less plastic than generalists [4]. Thus, where species abundance and the quality and availability of resources has been distorted, such as in British woodlands, the impact of elevated interspecific competition on habitat specialists could be especially acute [5,64].

Although marsh tits may have the capacity to reduce competition on a temporal basis, they appear to have a limited ability to spatially avoid large groups of heterospecifics in environments such as Wytham Woods. Contrary to our expectations and supported by other studies [17,18,57], the feeding stations that attracted larger groups of heterospecifics were also visited by larger groups of marsh tits. This positive relationship between group sizes probably reflected higher densities of all tit species in the best available habitat, such as richer areas of mature broadleaf woodland [33,36,65]. Thus, as habitat specialists, marsh tits could be spatially constrained in limiting interference competition from the dominant sympatric species, so temporal partitioning and caching food may be the only means of avoiding negative interactions.

Caching food may also allow marsh tits to secure a proportion of an ephemeral resource that might otherwise be depleted by other species, giving them an advantage over the non-caching blue and great tits in respect of exploitation competition. However, with a limited availability of food in woods, the more mobile blue and great tits can still exploit supplementary food resources elsewhere [6,7,37]. These dispersed resources are unavailable to sedentary specialists like marsh tits or willow tits *Poecile montanus*, which are ecologically similar species that are both declining in Britain [66]. Consequently, the widespread provisioning of supplementary food in British gardens can benefit blue and great tits but not the resident specialists, inflating the abundance of the former [7,66,67]. At an elevated national abundance of dominant blue and great tits, far exceeding that observed in a temperate primeval woodland [2,3], subordinate specialists may be increasingly limited in their ability to avoid competition for food with the socially and numerically dominant heterospecifics [28,29,68].

Interspecific competition between dominant generalists and subordinate specialists may be a more widespread phenomenon in relation to a numerical imbalance between species [4,6]. For example, agricultural intensification and forest exploitation have reduced the abundance of invertebrates, fruits, seeding plants [69,70] and cavity-bearing trees [71], limiting the availability of key resources. While this impoverishment of food and ecological niches can have a direct negative impact on specialists, generalist consumers can still thrive in modified conditions, e.g. by switching to different natural or anthropogenic resources [4,5,37,72]. Thus, as potentially with marsh tits or willow tits [66,67], elevated populations of dominant sympatric and generalist heterospecifics may intensify interference competition for subordinate specialists, with possible negative consequences for their populations [1,5,32].

Further research on the survival of individuals of subordinate species under differing environmental conditions would be worthwhile to detect the thresholds at which interspecific competition may affect population dynamics [7]. The large sample sizes in the current study enabled us to detect patterns which might be more obscure if the study involved fewer individuals and smaller spatial and temporal scales. Also, the patterns we present are, admittedly, based on correlations, so experimental studies that manipulate access to food or species densities would be helpful to better infer causal relationships between interference and temporal foraging activity of subordinate and dominant species. Nevertheless, our study provides important empirical evidence indicating a significant negative effect of dominant generalists (blue and great tits) on the foraging behaviour of a subordinate specialist (marsh tits). The results may have wider applicability as an example of a subordinate specialist that is subject to increasing competition, with relevance for species conservation [4,32].

## Data Availability

The data are provided in electronic supplementary material [73].
